# GeographicalDifference, Rural-urban Transition and Trend in Stroke Prevalence in China: Findings from a National Epidemiological Survey of Stroke in China

**DOI:** 10.1038/s41598-019-53848-1

**Published:** 2019-11-22

**Authors:** Xiaojuan Ru, Wenzhi Wang, Haixin Sun, Dongling Sun, Jie Fu, Siqi Ge, Limin Wang, Linhong Wang, Bin Jiang

**Affiliations:** 1 Beijing Neurosurgical Institute, Beijing Tiantan Hospital, Capital Medical University, Beijing, China; 2 Beijing Municipal Key Laboratory of Clinical Epidemiology, Beijing, China; 3 National Center for Chronic and Non-communicable Disease Control and prevention, Chinese Center for Disease Control and prevention, Beijing, China

**Keywords:** Cerebrovascular disorders, Stroke

## Abstract

Accurate and up-to-date provincial and regional-level stroke prevalence estimates are important for research planning and targeted strategies for stroke prevention and management. However, recent and comprehensive evaluation is lacking over the past 30 years in China. This study aimed to examine the geographical variations in stroke prevalence based on data from the National Epidemiological Survey of Stroke in China (NESS-China) and demonstrate urban-rural transition and trend over three decades. The stroke prevalence (prevalence day, August 31, 2013) was estimated using the world standard population. The stroke prevalence was 873.4 per 100,000 population, and varied from 218.0 in Sichuan to 1768.9 in Heilongjiang. Stroke prevalence exhibited a noticeable north-south gradient (1097.1, 917.7, and 619.4 in the north, middle, and the south, respectively; P < 0.001) and showed a 2.0-fold, 1.5-fold, and 1.2-fold increase in rural areas in the north, the middle, and the south, respectively, from 1985 to 2013. Overall, stroke prevalence was higher in the rural regions than in the urban (945.4 versus 797.5, P < 0.001) regions. However, the converse was depicted in 12 provinces. A noticeable geographical variation in stroke prevalence was observed and was evolving overtime in China. It is imperative that effective public health policies and interventions be implemented, especially in those regions with higher prevalence.

## Introduction

Stroke is the second leading cause of death and the third leading cause of disability-standardized life-years lost globally^1^. It is estimated that over two-thirds of stroke deaths worldwide occur in developing countries^2^. In China, stroke burden has increased over the past 30 years, in both the rural and urban population, with 2.4 million new strokes and 1.1million stroke-related deaths each year; presently, there are over 11.1 million stroke survivors^3^.

Some studies^1,2,4,5^ have reported substantial geographic variations in the distribution of stroke globally. The differences exist not only between countries but also between regions within a country. High-stroke incidence, mortality, and morbidity were identified in Eastern Europe, Eastern and Southeastern Asia, Central Africa, and Oceania^1^. In the1960s, high stroke mortality in the Southeastern United States(the so-called Stroke Belt), especially along the coasts of Georgia and the Carolinas (so-called Stroke Buckle), was reported^6^. As Mehndiratta *et al*.^4^ reportedthat Asia is home toa very diverse population both in terms of ethnic variability and socioeconomic difference, with regions in various stages of development and epidemiological transition. The same is true in China. The prevalence of stroke, therefore, is also expected to exhibit geographical differences. At present, the available data on geographical differences of stroke prevalence in China are from two surveys (the 6-city stroke study and the 22 rural population study) conducted 30 years ago. The study reported a north-south gradient with a significantly higher incidence, prevalence, and mortality of stroke in the north compared to that in the south^7,8^. Xu *et al*. reported the existence of a stroke belt with a high stroke incidence in nine provincial regions of northern and western China^9^.

In the past 30 years, China has experienced a rapid socioeconomic development, including an increase in the aging population, increased gross national product, and a change in lifestyle factors. As a result, epidemiological features of stroke may have been undergoing a significant change. Data on geographical differences in stroke prevalence need regular updating to facilitate health care planning and resource allocation. Current, updated, and accurate data are urgently needed. We aimed to examine the geographical variations in stroke prevalence at the country level based on data from the National Epidemiological Survey of Stroke in China (NESS-China) and demonstrate urban-rural transition and trend over three decades.

## Results

### Participant characteristics

A total of 596,536 individuals were screened. Of these, 50.3% were male, 42.2% were <35 years of age, and 11.9% were ≥65 years of age. The mean age was 39.0 years for male and 40.2 years for female. About 8.7% of the participants had a college and higher degree, and 65.6% were married or living with a partner. The characteristics of the participants are summarized in Table 1.

**Table 1:** Characteristics of the study participants.

Characteristics	Male	Female	Overall
Participants, n (%)	300192 (50.3)	296344 (49.7)	596536 (100.00)
Age Mean ± SD	39.0 ± 20.6	40.2 ± 20.6	39.6 ± 20.6
Age group, n (%)			
<35	128973 (43.0)	122844 (41.5)	251817 (42.2)
35–45	50759 (16.9)	48823 (16.5)	99582 (16.7)
45–55	46879 (15.6)	46884 (15.8)	93763 (15.7)
55–65	39366 (13.1)	40789 (13.8)	80155 (13.4)
65–75	21902 (7.3)	22938 (7.7)	44840 (7.5)
>75	12313 (4.1)	14066 (4.7)	26379 (4.4)
Education, n (%)			
Primary school or lower	115885 (38.6)	133031 (44.8)	248196 (41.7)
Middle school	156477 (52.1)	137732 (46.5)	294209 (49.3)
College and higher	26886 (9.0)	24844 (8.4)	51730 (8.7)
Missing	944 (0.3)	737 (0.2)	1681 (0.3)
Marital status, n (%)			
unmarried	65912 (22.0)	50905 (17.2)	116817 (19.6)
Married	193626 (64.5)	197534 (66.7)	391160 (65.6)
Divorced and Widowed	11235 (3.7)	21499 (7.3)	32734 (5.5)
Not applicable	27827 (9.3)	25006 (8.4)	52833 (8.9)
Missing	1592 (0.5)	1400 (0.5)	2992 (0.5)

SD, standard deviation;

### Provincial-level variations

**Figure 1:**
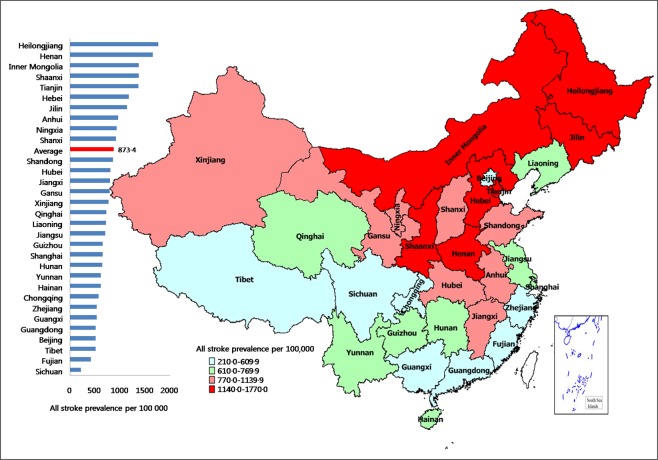
Map of China showing the prevalence of total (all types) stroke.

Table 2 shows the crude and age-standardized stroke prevalence. All 7,679 participants were diagnosed with stroke. The overall age-standardized prevalence of stroke was 873.4 per 100,000 population. There were remarkable provincial-level differences in the distribution of stroke prevalence. The age-standardized prevalence of total (all types) stroke (per 100,000 population) was the highest in Helongjiang (1768.9, 95%CI: 1735.4–1802.3), followed by Henan (1660.5, 95%CI: 1628.1–1693.0) Inner Mongolia (1380.6, 95%CI: 1351.0-1410.2) and Shaanxi (1375.2, 95%CI: 1345.7–1404.8). Sichuan had the lowest prevalence of total stroke (218.0, 95%CI: 206.2–229.9), followed by Fujian (412.6, 95%CI: 396.3–428.9) and Tibet (508.2, 95%CI: 490.1–526.2). A more than 8 times difference in stroke prevalence occurred between the highest and the lowest provincial regions (Fig. 1).

**Table 2:** Prevalence of stroke per 100,000 population (with 95%CI) in the 31 provincial regions.

Province	Crude prevalence			Age-standardizedprevalence		
	All stroke	IS	HS	All stroke	IS	HS
Anhui	1490.4 (1459.6–1521.1)	1123.7 (1096.9–1150.4)	335.1 (320.5–349.8)	965.5 (940.7–990.3)	721.9 (691.7–752.2)	223.5 (206.6–240.4)
Beijing	951.4 (926.7–976.0)	777.2 (754.9–799.5)	174.2 (163.6–184.8)	509.9 (491.8–527.9)	423 (399.8–446.2)	86.9 (76.3–97.4)
Chongqing	981.7 (956.7–1006.8)	745.6 (723.8–767.5)	236.1 (223.8–248.4)	570.7 (551.6–589.8)	425.4 (402.1–448.7)	145.3 (131.7–158.9)
Fujian	519.8 (501.6–538.1)	355.4 (340.3–370.5)	111.4 (102.9–119.9)	412.6 (396.3–428.9)	280.5 (261.6–299.4)	89.1 (78.4–99.8)
Gansu	951.2 (926.6–975.8)	793.6 (771.1–816.1)	157.6 (147.5–167.7)	795.0 (772.5–817.6)	672.6 (643.4–701.9)	122.4 (109.9–134.9)
Guangdong	690.6 (669.6–711.6)	507.7 (489.6–525.7)	160.1 (149.9–170.2)	512.2 (494.1–530.4)	379.4 (357.4–401.4)	117.5 (105.3–129.8)
Guangxi	689.9 (668.9–711.0)	473.1 (455.7–490.5)	212.9 (201.2–224.60	533.9 (515.4–552.4)	366.4 (344.8–388.0)	164.4 (149.9–178.9)
Guizhou	738.0 (716.2–759.7)	480.9 (463.3–498.4)	214.2 (202.5–226.0)	653.3 (632.8–673.7)	427.1 (403.8–450.4)	189 (173.4–204.5)
Hainan	817.9 (795.0–840.7)	766.8 (744.6–788.9)	51.1 (54.4–56.9)	614.8 (595.0–634.7)	576.4 (549.3–603.5)	38.4 (31.4–45.4)
Hebei	1593.9 (1562.2–1625.7)	1305.1 (1276.3–1333.9)	275.3 (262.0–288.6)	1180.5 (1153.1–1207.9)	970.2 (935.2–1005.3)	200.7 (184.7–216.7)
Henan	2691.3 (2650.3–2732.4)	2138.9 (2102.2–2175.6)	501.0 (483.1–518.9)	1660.5 (1628.1–1693.0)	1313.0 (1272.3–1353.7)	311.7 (291.7–331.6)
Heilongjiang	2499.1 (2459.4–2538.7)	2159.3 (2122.4–2196.2)	305.8 (291.8–319.8)	1768.9 (1735.4–1802.3)	1541.7 (1497.7–1585.8)	205.0 (188.8–221.2)
Hubei	1371.4 (1341.8–1400.9)	1092.7 (1066.3–1119.1)	278.6 (265.2–292.0)	804.7 (782.1–827.4)	638.9 (610.4–667.4)	165.8 (151.3–180.4)
Hunan	908.7 (884.6–932.8)	552.8 (534.0–571.6)	333.2 (318.6–347.8)	643.1 (622.8–663.4)	391.2 (368.9–413.6)	230.5 (213.5–247.7)
Jilin	2108.2 (2071.8–2144.7)	1731.2 (1698.1–1764.3)	339.9 (325.1–354.6)	1141.2 (1114.3–1168.2)	916.2 (882.1–950.3)	182.3 (167.1–197.6)
Jiangsu	1173.5 (1146.1–1200.8)	874.0 (850.3–897.6)	299.5 (285.7–313.4)	705.4 (684.2–726.7)	524 (498.1–549.8)	181.5 (166.3–197.6)
Jiangxi	1013.8 (988.4–1039.2)	735.9 (714.2–757.6)	262.5 (249.5–275.4)	798.2 (775.6–820.8)	577.6 (550.5–604.7)	208.3 (192.0–224.6)
Liaoning	1174.8 (1147.5–1202.2)	982.8 (957.7–1007.8)	169.7 (159.3–180.2)	718.3 (696.9–739.8)	599.6 (572.0–627.2)	103.8 (92.3–115.3)
Inner Mongolia	1780.3 (1746.7–1813.8)	1366.6 (1337.2–1396.1)	350.8 (335.8–365.8)	1380.6 (1351.0–1410.2)	1075.1 (1038.2–1112.0)	259.8 (241.6–278.0)
Ningxia	1102.6 (1076.1–1129.1)	871.8 (848.2–895.4)	230.8 (218.6–242.9)	931.1 (906.7–955.4)	743.5 (712.8–774.2)	187.6 (172.1–203.1)
Qinghai	880.0 (856.3–903.8)	563.2 (544.2–582.2)	299.2 (285.4–313.1)	724.0 (702.5–745.5)	463.7 (439.4–488.0)	246.5 (228.7–264.2)
Shandong	1526.7 (1495.6–1557.8)	1140.0 (1113.1–1166.9)	336.7 (322.0–351.4)	860.8 (837.4–884.3)	634.3 (605.9–662.7)	193.8 (178.1–209.5)
Shanxi	1290.7 (1262.0–1319.3)	1006.6 (981.2–1031.9)	284.1 (270.6–297.6)	922.0 (897.8–946.3)	717.5 (687.4–747.7)	204.5 (188.3–220.6)
Shaanxi	1764.6 (1731.2–1798.1)	1402.8 (1373.0–1432.6)	257.8 (244.9–270.6)	1375.2 (1345.7–1404.8)	1100.3 (1063.0–1137.6)	195.4 (179.6–211.2)
Shanghai	1652.9 (1620.5–1685.2)	1356.9 (1327.5–1386.2)	296.0 (282.3–309.8)	650.5 (630.1–670.9)	529 (503.0–554.9)	121.6 (109.1–134.0)
Sichuan	333.9 (319.2–348.5)	213.8(202.1–225.5)	108.8 (100.4–117.2)	218.0 (206.2–229.9)	138.2 (124.9–151.5)	73 (63.4–82.7)
Tianjin	2782.3 (2740.5–2824.0)	2395.5 (2356.7–2434.3)	386.8 (371.0–402.5)	1367.1 (1337.6–1396.5)	1181.5 (1142.8–1220.1)	185.6 (170.2–201.0)
Tibet	465.9 (448.7–483.2)	100.7 (92.7–108.8)	239.3 (226.9–251.7)	508.2 (490.1–526.2)	93.5 (82.0–104.5)	249.2 (231.4–267.0)
Yunnan	797.5 (774.9–820.1)	613.5 (593.7–633.3)	175.3 (164.7–185.9)	616.0 (596.2–635.9)	633.3 (604.9–661.7)	117.9 (105.6–130.2)
Zhejiang	911.2 (887.1–935.3)	659.6 (639.0–680.1)	212.6 (200.9–224.3)	536.0 (517.5–554.6)	470.5 (446.0–494.9)	139.3 (126.0–152.6)
Xinjiang	1170.8 (1143.5–1198.1)	968.6 (943.8–993.5)	168.5 (158.1–178.9)	772.9 (750.7–795.2)	387.9 (365.7–410.2)	125.8 (113.1–138.5)
Total	1287.3 (1258.7–1315.9)	999.8 (974.6–1025.0)	260.5 (247.6–273.4)	873.4 (849.8–897.0)	676.7 (647.4–706.1)	178.3 (163.2–193.4)

Age-standardized prevalence was standardized to the WHO world standard population.

CI: confidence interval; IS: ischemic stroke; HS: hemorrhagic stroke.

**Figure 2:**
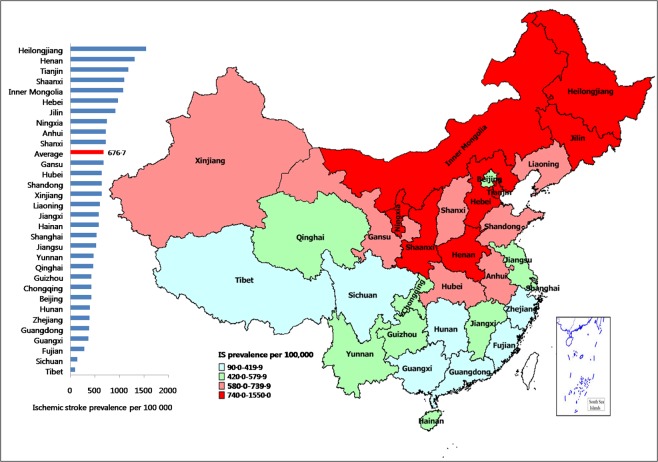
Map of China showing the prevalence of ischemic stroke.

The ischemic stroke age-standardized prevalence was 676.7 per 100,000 population,and was highest in Helongjiang (1541.7, 95% CI: 1497.7–1585.8), followed by Henan (1313.0, 95% CI: 1272.3–1353.7) and Tianjin (1181.5, 95% CI: 1142.8–1220.1). Tibet had the lowest prevalence of ischemic stroke per 100,000 population (93.5, 95%CI: 82.0–104.5), followed by Sichuan (138.2, 95% CI: 124.9–151.5). A more than 16 times difference in ischemic stroke prevalence occurred between the highest and the lowest provincial regions. Unlike the rank in the prevalence of overall stroke (Shaanxi ranked third), Tianjin ranked third in the prevalence of ischemic stroke (Table 2, Fig. 2).

**Figure 3:**
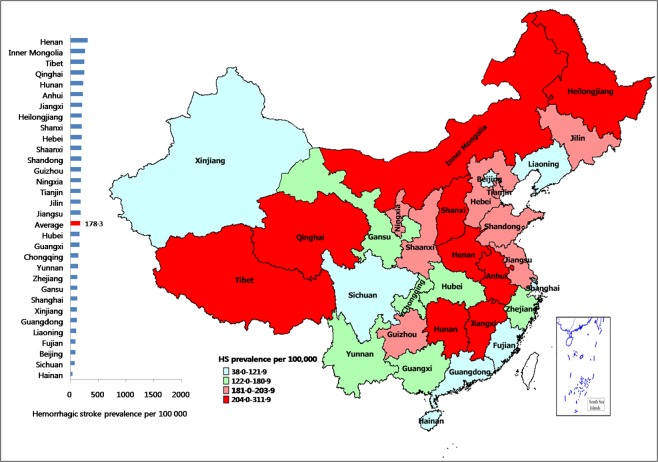
Map of China showing the prevalence of hemorrhagic stroke.

The hemorrhagic stroke age-standardized prevalence (per 100,000 population) was 178.3. The age-standardized hemorrhagic stroke prevalence was highest in Henan (311.7, 95%CI: 291.7–331.6), followed by Inner Mongolia (259.8, 95%CI: 241.6–278.0) and Tibet (249.2, 95%CI: 231.4–267.0). Hainan had the lowest prevalence of hemorrhagic stroke (38.4, 95%CI: 31.4–45.4), followed by Sichuan (73.0, 95%CI: 63.4–82.7) (Table 2, Fig. 3).

The prevalence of ischemic stroke (676.7) was significantly higher than that of hemorrhagic stroke (178.3). The ratio of the prevalence for the two types of stroke ranged from 2.4 to 4.9, and averaged 3.8. However, there was an exception; Tibet had a higher prevalence of hemorrhagic stroke (249.2) than ischemic stroke (93.5) (Table 2).

### Regional-level variations

Withthe Yellow River and Yangtze river acting as the boundary, the total stroke age-standardized prevalence (per 100,000 population) was 1097.1(95%CI: 1059.8–1134.3) in the north of the Yellow River, 917.7(95%CI: 883.6–951.8) in the middle, and 619.4(95%CI: 591.3–647.4) in the south of the Yangtze River; exhibiting a north-south gradient. There were significant differences in the distribution of total stroke prevalence between different regions (P < 0.001) (Table 3). Compared to the rural study in 22 provincial regions in 1985^10^, there was a significant increase of stroke prevalence (age-standardized to the 1960 United States population) in rural areas, including a 2.0-fold increase in the north (from 480.0 to 1433.1), a 1.5-fold increase in the middle (from 420.0 to 1051.4) and a 1.2-fold increase in the south (from 280.0 to 623.1) (Supplemental Fig. 1).

**Table 3:** The prevalence of stroke types per 100,000 population (with 95%CI)in different regions.

Regions	Crude prevalence			Age-standardized prevalence		
	All stroke	IS	HS	All stroke	IS	HS
North	1606.6 (1574.7–1638.5)	1317.8 (1288.9–1346.7)	267.1 (254.0–280.1)	1097.1 (1059.8–1134.3)	900.8 (867.0–934.6)	181.6 (166.4–196.9)
Middle	1452.0 (1421.6–1482.3)	1116.5 (1089.9–1143.1)	298.5 (284.6–312.3)	917.7 (883.6–951.8)	703.1 (673.2–733.0)	190.6 (175.1–206.3)
South	884.3 (860.5–908.1)	630.0 (609.9–650.1)	229.2 (217.1–241.3)	619.4 (591.3–647.4)	438.7 (415.1–462.4)	163.2 (148.8–177.7)
χ^2^	497.809	556.618	17.580	300.358	356.669	4.290
p	<0.001	<0.001	<0.001	<0.001	<0.001	0.117
Eastern	1205.3 (1177.6–1233.0)	948.1 (923.5–972.7)	233.4 (221.2–245.7)	754.6 (723.6–785.6)	592.5 (565.0–619.9)	146.6 (132.9–160.3)
Central	1713.0 (1680.1–1745.9)	1350.4 (1321.1–1379.7)	337.5 (322.8–352.2)	1124.3 (1086.6–1162.0)	883.9 (850.4–917.4)	223.9 (207.0–240.8)
Western	953.9 (929.3–978.6)	709.3 (688.0–730.6)	212.6 (200.9–224.3)	731.0 (700.6–761.5)	543.2 (516.9–569.5)	163.9 (149.4–178.3)
χ^2^	463.953	418.394	67.871	233.567	206.146	39.947
p	<0.001	<0.001	<0.001	<0.001	<0.001	<0.001

Age-standardized prevalence was standardized to the WHO world standard population.

IS:ischemic stroke; HS: hemorrhagic stroke; CI: confidence interval.

The age-standardized prevalence of ischemic stroke was 900.8 (95%CI: 867.0–934.6) in the north of the Yellow River, 703.1 (95%CI:673.2–733.0) in the middle, and 438.7(95%CI: 415.1–462.4) in the south of the Yangtze River. There were significant differences in ischemic stroke prevalence between different regions (P < 0.001)(Table 3).

The age-standardized prevalence of hemorrhagic stroke was 181.6(95%CI: 166.4–196.9) in the north of the Yellow River, 190.6(95%CI: 175.1–206.3) in the middle, and 163.2 (95%CI:148.8–177.7) in the south of the Yangtze River. There were no significant differences in hemorrhagic stroke prevalence between different regions (P = 0.117) (Table 3).

In the 3 regions divided by economic level, the total stroke age-standardized prevalence was highest in the central (1124.3, 95%CI: 1086.6–1162.0), followed by the eastern (754.6, 95%CI: 723.6–785.6) and western (731.0, 95%CI: 700.6–761.5). The age-standardized prevalence of ischemic stroke was 592.5 (95%CI: 565.0–619.9), 883.9 (95%CI: 850.4–917.4), and 543.2 (95%CI: 516.9–569.5) in the eastern, central, and the western, respectively. The age-standardized prevalence of hemorrhagic stroke was 146.6 (95%CI: 132.9–160.3), 223.9 (95%CI: 207.0–240.8), and 163.9 (95%CI:149.4–178.3)in the eastern, the central, and the western, respectively. Differences in the prevalence of total stroke, ischemic stroke, and hemorrhagic stroke in the 3 regions were statistically significant (P < 0.001) (Table 3).

### Rural-urban difference and transition

**Figure 4:**
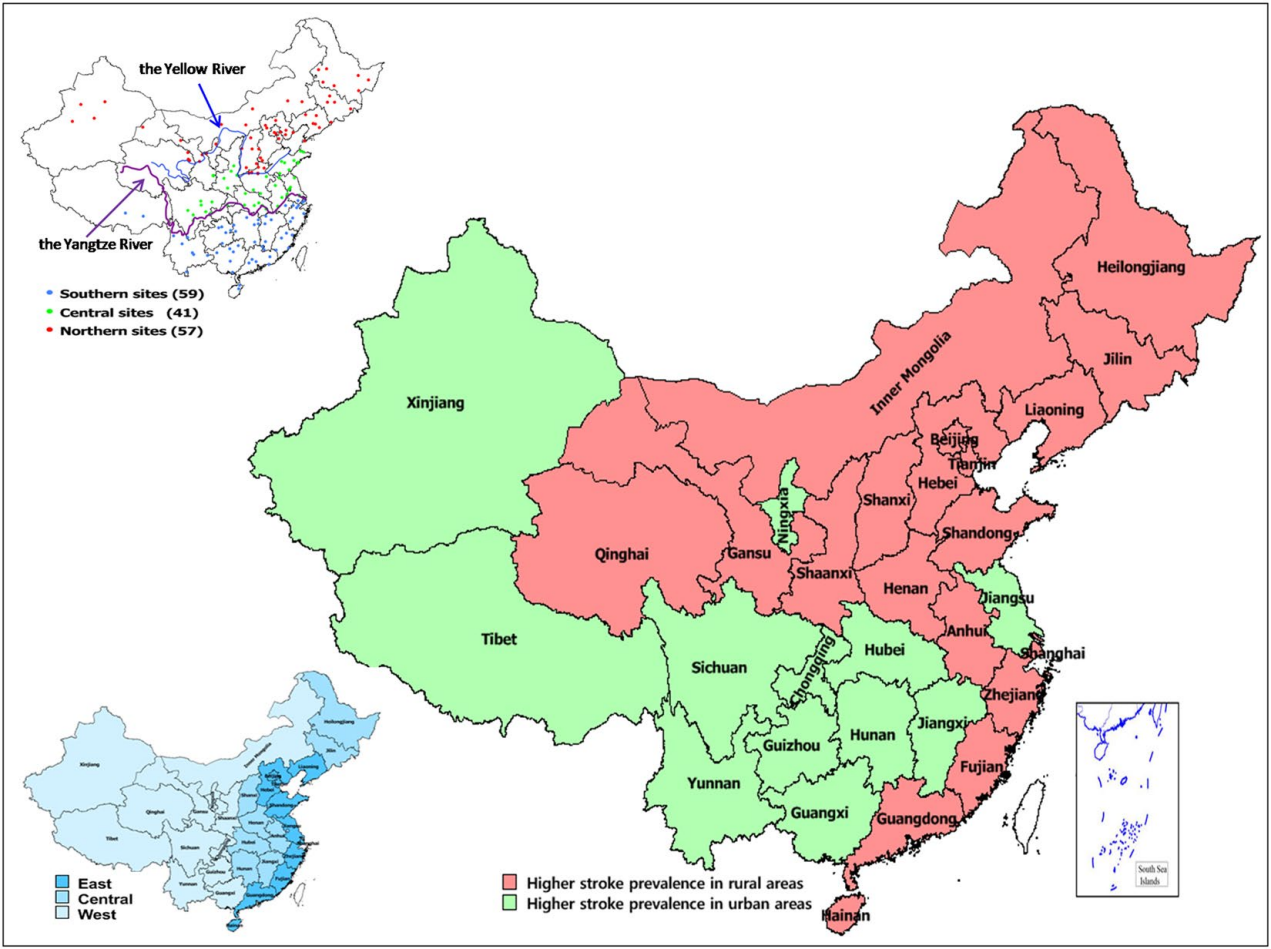
Map of China showing the distribution of higher stroke prevalence in rural and urban areas.

The observed stroke age-standardized prevalence (per 100,000 population) in the rural (945.4, 95%CI: 910.8–980.0) region was higher than that observed in the urban region (797.5, 95%CI: 765.7–829.3) (P < 0.001) (Table 4). However, in some provincial regions with similar epidemic characteristics since30 years, such as Chongqing, Guangxi, Guizhou, Hubei, Hunan, Jiangsu, Jiangxi, Ningxia, Sichuan, Tibet, Yunnan, and Xinjiang, the prevalence of stroke appeared to be higher in the urban than inthe rural areas(Table 4, Fig. 4). The prevalence of stroke across the 12 provinces was 708.5 (95%CI: 678.5–738.5) and 539.1 (95%CI: 512.9–565.2) in the rural and urban areas, respectively (Supplemental Table 1).

**Table 4:** The prevalence of stroke in rural and urban areas of the 31 provincial regions.

Province	Crude prevalence of all stroke		χ2	p	Age-standardized of all stroke		χ2	p
	Rural	Urban			Rural	Urban		
Anhui	1652.5 (1607.8–1697.1)	1245.9 (1205.0–1286.8)	6.845	<0.001	1015.2 (979.3–1051.1)	890.3 (856.7–923.9)	1.016	0.313
Beijing	1008.3 (973.3–1043.3)	904.4 (869.5–939.3)	2.212	0.645	590.1 (563.7–617.5)	440.0 (416.4–463.7)	0.856	0.355
Chongqing	933.1 (899.5–966.8)	1826.5 (1777.1–1875.8)	3.400	0.065	559.8 (533.1–586.5)	716.1 (694.7–737.5)	0.105	0.746
Fujian	682.6 (653.7–711.4)	343.1 (321.5–364.6)	10.486	0.001	597.2 (569.6–624.7)	245.7 (228.0–263.4)	14.063	<0.001
Gansu	982.6 (948.1–1017.1)	912.8 (877.8–947.9)	0.227	0.634	799.0 (767.2–830.9)	789.0 (757.4–820.7)	0.006	0.937
Guangdong	587.5 (560.8–614.3)	899.5 (864.7–934.3)	6.867	0.009	512.3 (486.7–537.8)	509.4(484.0–534.9)	0.001	0.997
Guangxi	493.3 (468.7–517.8)	816.6 (783.4–849.8)	9.221	0.002	433.6 (410.1–457.1)	587.6 (560.3–615.0)	2.831	0.092
Guizhou	461.9 (438.2–485.7)	986.8 (950.4–1023.2)	19.693	<0.001	435.7 (412.1–459.2)	841.1 (808.5–873.8)	13.692	<0.001
Hainan	882.1 (849.3–914.8)	751.9 (720.0–783.7)	0.409	0.527	662.7 (633.7–691.8)	542.0 (515.8–568.3)	0.402	0.526
Hebei	2027.4 (1978.1–2076.7)	1207.9 (1167.7–1248.2)	31.390	<0.001	1553.7 (1509.4–1597.9)	874.9 (841.6–908.2)	28.543	<0.001
Henan	2279.4 (2227.1–2331.6)	3238.8 (3173.6–3304.1)	26.816	<0.001	1648.5 (1602.9–1694.0)	1643.9 (1598.4–1689.4)	23.751	<0.001
Heilongjiang	2816.0 (2758.1–2873.9)	2206.6 (2152.4–2260.7)	10.079	0.002	2198.8 (2146.4–2251.3)	1442.7 (1400.1–1485.4)	1.871	0.171
Hubei	1078.3 (1042.1–1114.4)	1598.0 (1551.8–1644.2)	11.279	0.001	756.2 (725.3–787.2)	821.5 (789.2–853.8)	0.254	0.614
Hunan	877.5 (844.8–910.1)	950.7 (914.9–986.4)	0.384	0.535	613.4 (585.5–641.4)	683.0 (635.5–712.4)	0.503	0.478
Jilin	2238.7 (2197.2–2290.7)	2063.8 (2011.4–2116.1)	0.530	0.466	1180.6 (1141.9–1219.2)	1133.7 (1095.9–1171.6)	20.212	<0.001
Jiangsu	1034.1 (998.7–1069.5)	1302.2 (1206.4–1344.0)	3.770	0.052	653.8 (623.9–681.6)	755.0 (724.0–786.0)	1.015	0.314
Jiangxi	847.3 (815.2–879.4)	1070.8 (1032.9–1108.7)	1.838	0.175	726.7 (696.3–757.1)	823.6 (791.3–855.9)	0.429	0.513
Liaoning	1396.5 (1355.4–1437.5)	577.7 (549.7–605.6)	25.518	<0.001	884.7 (851.2–918.2)	324.9 (304.5–345.3)	0.072	0.789
InnerMongolia	2239.0 (2187.2–2290.7)	1320.8 (1278.7–1362.8)	23.021	<0.001	1784.9 (1737.5–1832.3)	969.5 (934.4–1004.5)	25.189	<0.001
Ningxia	975.6 (941.2–1010.0)	1154.2 (1114.8–1193.6)	0.469	0.494	899.1 (865.3–932.8)	926.9 (892.6–961.1)	0.019	0.89
Qinghai	728.7 (698.9–758.5)	908.2 (873.3–943.2)	0.556	0.456	837.8 (805.2–870.4)	685.8 (656.2–715.3)	0.49	0.484
Shandong	1894.6 (1846.9–1942.4)	1056.0 (1018.3–1093.7)	34.562	<0.001	1096.7 (1059.4–1133.9)	576.2 (549.1–603.3)	0.365	0.546
Shanxi	1935.3 (1887.1–1983.5)	820.9 (787.7–854.2)	58.559	<0.001	1445.1 (1402.4–1487.8)	574.9 (547.8–601.9)	20.765	<0.001
Shaanxi	1774.3 (1728.1–1820.5)	1681.1 (1633.7–1728.4)	0.094	0.760	1392.8 (1350.9–1434.7)	1312.5 (1271.8–1353.2)	10.496	0.001
Shanghai	2241.4 (2189.6–2293.3)	1383.9 (1340.9–1426.9)	7.896	0.005	688.8 (659.2–718.4)	632.7 (604.3–661.0)	0.167	0.683
Sichuan	333.8 (313.6–354.0)	333.9 (312.7–355.2)	0.001	0.994	204.4 (188.3–220.6)	236.6 (219.2–254.0)	0.272	0.602
Tianjin	3239.3 (3177.3–3301.3)	2364.5 (2308.5–2420.4)	5.658	0.017	2186.4 (2134.1–2238.7)	880.6 (847.2–914.0)	23.104	<0.001
Tibet	463.6 (439.9–487.4)	510.2 (484.0–536.5)	6.706	0.010	505.9 (480.5–531.2)	750.6 (719.7–781.5)	0.498	0.48
Yunnan	694.9 (665.8–724.0)	950.8 (915.1–986.6)	4.538	0.033	530.3 (504.3–556.3)	738.1 (707.5–768.7)	4.214	0.04
Zhejiang	852.7 (820.6–884.9)	993.7(957.2–1030.3)	1.232	0.267	519.0 (493.3–544.7)	564.7 (537.9–591.5)	0.219	0.64
Xinjiang	802.1 (770.9–833.4)	2003.3 (1951.7–2054.9)	31.490	<0.001	707.7 (677.7–737.7)	829.2 (796.8–861.6)	0.481	0.488
Total	1322.4 (1282.4–1362.4)	1248.3 (1207.4–1289.2)	6.431	0.011	945.4 (910.8–980.0)	797.5 (765.7–829.3)	42.7	<0.001

Age-standardized prevalence of all stroke was standardized to the WHO world standard population.

Compared tothe rural study in 22 provincial-regions in 1985^7^, in the past 3 decades, rural areas in these regions have experienced a greater increase in stroke prevalence (from 393.7 to 1014.7). The prevalence of stroke has increased most significantly by 5.3, 3.9, 3.3, and 3.1 times in Inner Mongolia,Guangxi, Tianjin, and Hubei, respectively. The minimum increase was found in Sichuan (from 210.4 to 219.1), Hunan (from 432.5 to 652.6), and Guizhou (from 267.1 to 456.7) (Supplemental Fig. 2).

Compared to the urban study in 6 cities in 1983^8^, the prevalence of stroke in urban areas has increased slightly over the past 3 decades (from 719.0 to 816.2). Stroke prevalence increased in urban areas of Heilongjiang (from 1249.0 to 1552.3), Ningxia (from 824.0 to 974.9), and Shanghai (from 615.0 to 693.6), while it decreased in urban areas of Sichuan (from 456.0 to 257.0), Hunan (from 732.0 to 846.0), and Guangdong (from 576.0 to 552.0) (Supplemental Fig. 2).

The stroke age-standardized prevalence was higher in rural areas than in urban areas in the various regions surveyed, except for the southern regions (rural, 580.9; urban, 657.6) (Supplemental Table 1).

## Discussion

The present study, which differs from the stroke registries, cohort studies, or reviews, is the first to report stroke prevalence across the 31 provincial regions based on a large nationwide representative survey for all ages. Although the estimates from previous studies^7,10^ showed that there were significant geographical differences in stroke prevalence, our analysis provided a more detailed insight into the prevalence of stroke, with rural-urban transition, and the trend in the past three decades. Most studies^9,11^ in China have shown geographical variations in stroke incidence and mortality. However, provincial and regional level stroke prevalence estimates for all ages are not available. In the earlier 22 rural population study^7^, the age-standardized stroke prevalence (age-standardized to the 1960 total US population)was 393.7, and the highest stroke prevalence was 869.6 in Heilongjiang and lowest (95.4)in Guangxi. In the 6-city study^8^, the age-standardized stroke prevalence (age-standardized to the 1960 total US population) was 719.0, with the highest (909.0) in Harbin of Heilongjiang and the lowest in Shanghai.

In Germany, a population-based survey of 28,090 participants (aged ≥50 years) reported an increased stroke prevalence to 7.6% (men 8.4%; women 7.2%). Factors associated with the higher prevalence were increasing age, sex (male), non-German nationality, lower education, positive family history of stroke, and solitary living^12^. In Urban Sri Lanka, a population-based, cross-sectional study conducted among 2,313 adults aged ≥18 years found a stroke prevalence of 10.4 per 1,000 (95%CI: 6.3–14.5)^13^. In the present analysis, stroke prevalence was 873.4 per 100,000 population for all ages, and varied substantially among different Chinese populations in different regions. The provincial difference in stroke prevalence was also striking and varied from 218.0 in Sichuan province to 1768.9 in Heilongjiang. It was in general, higher in the north provincial region than in the south. However, for some provincial regions with high-income, such as Beijing in north China and the eastern and southern coastal provinces, the prevalence of stroke was relatively low. Adequate health services and strategies for stroke prevention and care (including blood pressure control, smoking cessation, and acute stroke units) are the most likely explanations for the lower stroke prevalence shown. Our study found that the high provincial prevalence of stroke was associated with a high provincial incidence of stroke (r = 0.905, p < 0.001), but was not related to the low provincial mortality (r = 0.185, p = 0.320).

Stroke prevalence exhibited a noticeable north-south gradient, which is consistent with the results of a previous study^10^. High stroke prevalence regions were mainly located in the north of the Yellow River, while low stroke prevalence regions are mainly in the south of the Yangtze River. The striking variation in stroke prevalence could be explained by the difference in the prevalence of hypertension between the northern and southern provincial regions. The China hypertension survey found geographical gradients in the prevalence pattern of hypertension, which is highest in northeast, north, and southwest China^14,15^. He *et al*. suggested that the geographic difference in stroke and hypertension in China is consistent with the “salt hypothesis,” which proposes that high salt intake increases both the risk of hypertension as well as stroke^11^. Since hypertension remains the most important risk factor for all types of stroke, we therefore retrieved the hypertension prevalence for 31 provincial regions from a review^9^ to analyze the correlation with stroke prevalence. We observed that the prevalence of hypertension correlated significantly with all types of stroke prevalence (r = 0.415, p = 0.020) and hemorrhagic stroke (r = 0.370, p = 0.041). The recent hypertension survey in China^16^ found that 23.2% (estimated 244.5 million) of the Chinese adult population aged ≥18 years had hypertension, while another 41.3% (estimated 435.3 million) had prehypertension. Among individuals with hypertension, 46.9% were aware of their condition, 40.7% were taking prescribed antihypertensive medications, and 15.3% had controlled hypertension. Therefore, hypertension, as one of the major risk factors for stroke, should be better managed. Studies^2,17^ have suggested that regions and country income levels are related to the burden of stroke, and the age-specific prevalence of stroke is significantly greater in high-income countries. Our study found that the central (middle-income) and eastern (high-income) regions showed higher prevalence of stroke compared to the western (low-income).

Stroke types also vary by country, regional income level, and ethnic differences^18,19^. A recent review of stroke epidemiology in the South, East, and South-east Asia reported that ischemic stroke occurs more commonly than hemorrhagic stroke, except in India and Vietnam, where the converse was observed^5^. According to previous studies in China^7,8,20,21^, ischemic stroke is the most common type of stroke. The proportion of stroke types varied from 43.7–78.9% for cerebral infarction to 18.8–47.6% for intracerebral hemorrhage^22^. The results of this study showed significantly higher ischemic stroke prevalence than that of hemorrhagic stroke. In general, it is more meaningful to use stroke incidence for classification. This is due to the survival of stroke patients, mostly mild or ischemic stroke patients. Patients with hemorrhagic stroke are generally more serious and have a higher proportion of deaths in the acute phase, thus, the higher prevalence of ischemic stroke compared to that of hemorrhagic stroke. However, there was an exception in Tibet, where the prevalence of hemorrhagic stroke was 2.7-fold higher than the prevalence of ischemic stroke. This finding may be explained as follows: (1) consideringthe fact that hypertension is the strongest contributor to hemorrhagic stroke, the highest hypertension prevalence (19.54%) occurred in Tibet^9^ and (2) there are only two survey sites included in Tibet, which might have influenced the findings.

With dramatic transformations in the social, economic, and environmental conditions over the past 30 years, the lifestyle in China has changed rapidly, especially in rural areas. As a result of an aging population, urbanization, and westernization, the main risk factors for stroke such as hypertension, diabetes mellitus, hypercholesterolemia and obesity have increased substantially, and resulted in a greater increase in stroke prevalence in rural areas than in urban areas for three decades. But the 12 provincial-level regions showed lower stroke prevalence in rural areas and 8 of them (Chongqing, Guangxi, Guizhou, Ningxia, Sichuan, Tibet, Yunnan, and Xinjiang) were in the western regions with underdeveloped economy.

The early stroke epidemiological surveys were mainly based on the clinical level and less dependent on the objective diagnostic methods. Along with the development of medical diagnosis, the use of brain CT or MRI in this study reached 89.4%, which guaranteed the reliability and accuracy of the results. The findings from our study should well reflect the current actual picture in China. The reasons for the difference in stroke prevalence in different regions are complex. These geographical differences may be attributed to the differences in risk factors, lifestyle, environmental, and sociocultural factors between Chinese populations. The specific reasons require further study.

## Limitations

There were some potential limitations to this study. First, the cross-sectional retrospective design is susceptible to recall bias, which resulted in the missing of some important information. However, repeated verification measures by the CDC preliminary screening and neurologists review minimized the recall bias. Secondly, the distribution of sampling sites was uneven. There were fewer sampling sites in the more populated regions or western China. Considering the operability, only two survey points were selected from Beijing and Shanghai with large populations, which may not have completely reflected the level of stroke prevalence. Compared to the study conducted 30 years ago, there was indeed a marked increase in stroke prevalence in the rural areas of China. The increase could be attributable to the markedly changed health behavior and lifestyles of people in rural areas with social and economic development. However, we should be cautious in interpreting the comparison results because of bias from different sampling methods in the two studies, as well as the recall bias. Thirdly, the data collected did not include risk factors, environmental, and sociocultural factors; thus, the relationships between stroke prevalence and these factors could not be established.

## Conclusions

The prevalence of stroke in China showed a noticeable geographical difference and was evolving overtime. The burden of stroke was increasingly severe and it is urgently needed to grasp provincial and regional level epidemiological data to better understand the burden of stroke. The comparison of stroke prevalence in different regions is helpful to understand the mechanism of stroke that is specific to risk factors, lifestyle, environmental, and sociocultural factors and also helpful to implement targeted secondary prevention measures. Further and better epidemiological studies should be needed to clarify the real reasons for the geographic differences. A better understanding of the reasons for these differences could guide public health prevention programs. The policies and interventions should target, especially those regions with higher stroke prevalence.

## Methods

### Study design and participants

We conducted an analysis on data derived from the NESS-China. The NESS-China was a nationally representative cross-sectional survey conducted in 157 sites of the 31 provincial regions in mainland China from September 1, 2013 to December 31, 2013. The 31 provincial regions included 22 provinces, 5 autonomous regions (Xinjiang, Ningxia, Tibet, Inner Mongolia, and Guangxi), and 4 municipalities (Beijing, Shanghai, Tianjin, and Chongqing). A multistage, stratified complex sampling method was used to select a nationally representative sample of all populations. The survey was conducted face-to-face by households. All the family members in a household were interviewed. For children younger than 14 years, older adults who had no articulate answers to the questions, and individuals with language barriers from disease or other reasons, the questions could be answered by other family members on their behalf. If a participant was absent (e.g., away on a business trip, study, or work), he/she could answer the questions by telephone with the help of a family member. Specific details on sample size calculation, sampling process, and participant selection for the NESS-China study have been published previously^3,23,24^. A total of 596,536 participants of all ages were included in our analysis.

### Data collection

The NESS-China was the first national survey to use the door-to-door and face-to-face data collectionmethod. The trained interviewers of the Center for Disease Control and Prevention (CDC) administered the preliminary screening tool to identify participants with positive stroke symptoms. A comprehensive questionnaire, which included family information on demographic characteristics, medical history, stroke-related symptoms and death information of family members, who died from stroke between September 1, 2012 and August 31, 2013. After the preliminary screening, participants with the symptoms or history suggestive of stroke were invited to see a neurologist in the town/village clinic of their choice. Their medical records (with information on e.g. cardiovascular disease risk factors, computed head tomography [CT], magnetic resonance imaging [MRI] scans, and autopsy protocols) were carefully reviewed and relevant data were recorded. When appropriate, some study participants were requested to undergo a brain-neuroimaging examination (e.g. to exclude brain disorders mimicking stroke) and another neurological examination.

**Figure 5:**
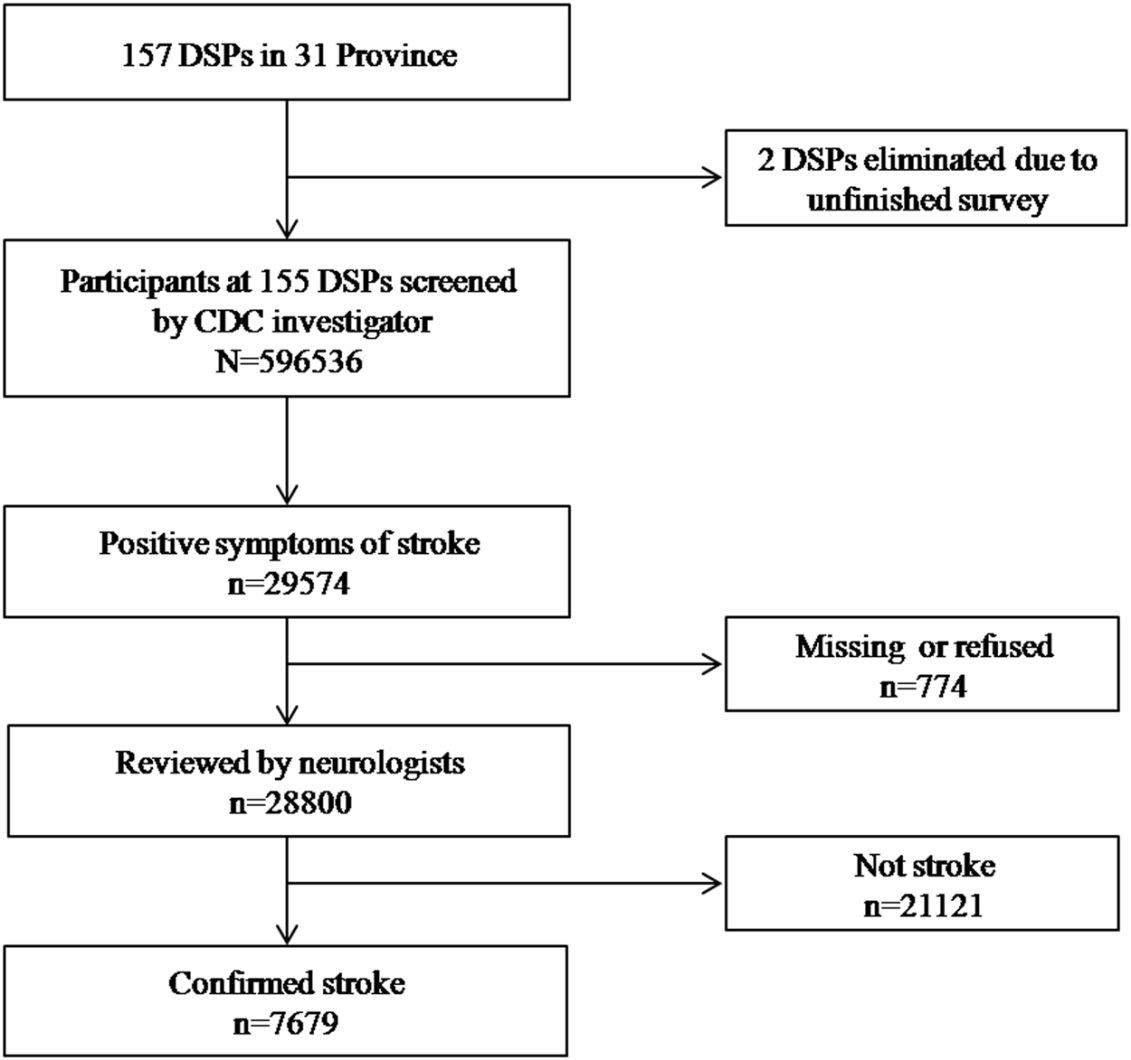
A flowchart for stroke case ascertainment.

Next, neurologists arranged face-to-face interviews with all confirmed and suspected cases. Based on the information obtained (e.g., history, neurological symptoms/signs, and imaging data such as CT and/or MRI), the neurologist would make a final diagnosis according to the stroke diagnostic criteria. Any participant who was not interviewed was contacted again to arrange for another interview. For complicated cases that could not be confirmed by the neurologist, the diagnosis confirmed on the basis of the discussion between two or more neurologists. If a diagnosis could still not be confirmed, relevant case data was documented and submitted to the provincial technical consultants for further consultation. Finally, a total of 28,800 participants with stroke or positive stroke symptoms were reviewed and 7,679 of these were confirmed as stroke by August 31, 2013. Two of the 157 sites failed to complete the investigation as required and were therefore excluded from the analysis (Fig. 5). Additional details on stroke confirmation can be found in the literature^3^.

The epidemiological data^7,8,10^ from two comparable studies (the 6-city stroke study and the 22 rural population study are similar to the present study in the methodology used for ascertaining stroke and the survey used) were used to confirm the urban-rural transition and trend over the three decades.

The study protocol was approved by the ethical review committees of Beijing Tiantan Hospital. All methods were performed in accordance with relevant guidelines and regulations. All participants provided written informed consent before data collection.

### Definitions

Based on the World Health Organization (WHO) criteria, stroke was defined as “rapidly developing clinical syndrome of focal (or global) abnormalities of cerebral function, lasting more than 24 hours or leading to death, with no apparent cause other than that of vascular origin^25^”. Any nervous system abnormalities induced by trauma, metabolic disorder, tumor, or central nervous system infections were excluded. All cases identified as definite stroke were further classified into ischemic, hemorrhagic, and undetermined stroke, and coded using the tenth revision of the international Classification of Diseases. The classification of ischemic and hemorrhagic stroke was performed on the basis of the clinical presentation, and confirmation by CT or MRI was required, with reference to the Atherosclerosis Risk in Communities criteria^26^. Undetermined stroke included all cases that did not have CT or MRI data or could not be classified by CT or MRI. The prevalence of stroke in this study was expressed as a point prevalence ratio (prevalence day, August 31, 2013).

With the Yellow River and Yangtze River acting as the boundary, the 157 survey sites were divided into three categories as follows: the north, the middle, and the south. The north of the Yellow River included 57 survey sites, the middle area between the Yellow River and the Yangtze River included 41 survey sites, and the south of the Yangtze River included 59 survey sites.

According to the level of economic development, the 31 provincial-regions were divided into three categories as eastern (11 provincial-regions including Beijing, Fujian, Guangdong, Hainan, Hebei, Jiangsu, Liaoning, Shandong, Shanghai, Tianjin, and Zhejiang), central(8 provincial-regions including Anhui, Henan, Heilongjiang, Hubei, Hunan, Jilin, Jiangxi, and Shanxi), and western (12 provincial-regions including Gansu, Guizhou, Ningxia, Qinghai, Shaanxi, Sichuan, Tibet, Xinjiang, Yunnan, Chongqing, Guangxi, and Inner Mongolia).

### Statistical analyses

With regard to sociodemographic factors, continuous variables were described as mean with standard deviations or as median (interquartile range [IQR]) according to their distribution. Categorical variables were reported as frequency (percent). All reported prevalence of stroke were estimated and age-standardized to the WHO world standard population^27^. For prevalence data, 95% confidence intervals (CIs) were calculated. When compared with the 6-city stroke study in 1983 and the 22 rural population study in 1985, stroke prevalence was standardized to the 1960 U.S. population. The comparison of prevalence or rates between different groups was performed by Chi-square test. Correlation analysis was used to identify the relationship between provincial stroke prevalence and provincial hypertension prevalence. All p values that were 2-tailed and <0.05 were considered statistically significant. All data analyses were conducted using SPSS for Windows version 13.0 (SPSS Inc., Chicago, IL, USA).

## Supplementary information

Supplemental Table 1, Supplemental Figure 1 and Supplemental Figure 2

## Data Availability

All data generated or analysed during this study are included in this published article (and its Supplementary Information Files).
