# Supplementary material for: GeographicalDifference, Rural-urban Transition and Trend in Stroke Prevalence in China: Findings from a National Epidemiological Survey of Stroke in China
Source: Sci Rep. 2019 Nov 22;9:17330. doi: 10.1038/s41598-019-53848-1 (PMC6874659; doi:10.1038/s41598-019-53848-1)
Supplement: Supplementary file 1 — Supplemental Table 1, Supplemental Figure 1 and Supplemental Figure 2 [file 41598_2019_53848_MOESM1_ESM.docx]

**Geographical Difference, Rural-urban Transition and Trend in Stroke Prevalence in China: Findings from a National Epidemiological Survey of Stroke in China**

Xiaojuan Ru, PhD ^1,2^, Wenzhi Wang, MD^1,2^, Haixin Sun, MD, PhD^1,2^, Dongling Sun, PhD ^1,2^,Jie Fu^1,2^,Siqi Ge,PhD ^1,2^, Limin Wang, MD ^3^, Linhong Wang, MD ^3*^, Bin Jiang, MD ^1,2*^,

^1^Beijing Neurosurgical Institute, Beijing Tiantan Hospital, Capital Medical University, Beijing, China

^2^Beijing Municipal Key Laboratory of Clinical Epidemiology, Beijing, China

^3^ National Center for Chronic and Non-communicable Disease Control and prevention, Chinese Center for Disease Control and prevention, Beijing, China

Supplemental Table 1. The prevalence of stroke in rural and urban areas of various regions

| Region | Prevalence of stroke | |
| --- | --- | --- |
|  | Rural | Urban |
| North, middle, and south regions | | |
| North | 1330.7(1289.7-1371.7) | 897.4(863.7-931.2) |
| Middle | 975.9(940.8-1011.1) | 833.5(801.0-866.0) |
| South | 580.9(553.7-608.1) | 657.6(628.7-686.6) |
| East, central, and west regions | |  |
| Eastern | 887.8(854.2-921.3) | 611.3(583.4-639.2) |
| Central | 1222.4(1183.1-1261.8) | 1039.7(1003.4-1076.0) |
| Western | 750.6(719.7-781.4) | 702.6(672.7-732.4) |
| Other Regions | | |
| Region 1 | 539.1(512.9-565.2) | 708.5(678.5-738.5) |
| Region 2 | 1168.1（1129.6-1206.5） | 848.3(815.5-881.1) |
| Total | 945.4（910.8-980.0） | 797.5(765.7-829.3) |

Region 1 included 12 provinces with higher stroke prevalence in urban areas, such as Jiangsu,Jiangxi, Hunan, Hubei, Chongqing, Guangxi, Guizhou, Ningxia, Sichuan, Tibet, Yunnan, and Xinjiang.

Region 2 included 19 provinces with higher stroke prevalence in rural areas, excluding the 12 provinces mentioned above.

**
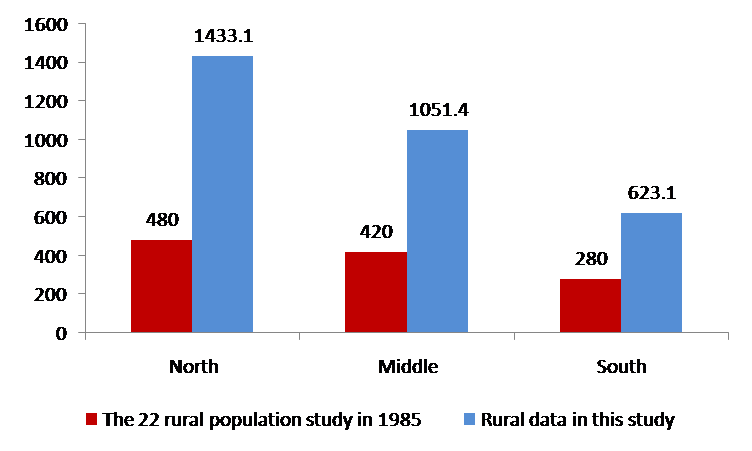
**

Supplemental Fig 1 Comparison of stroke prevalence (age-standardized to the 1960 United States population) between the rural data from this study and the rural data from the 1985 study

**
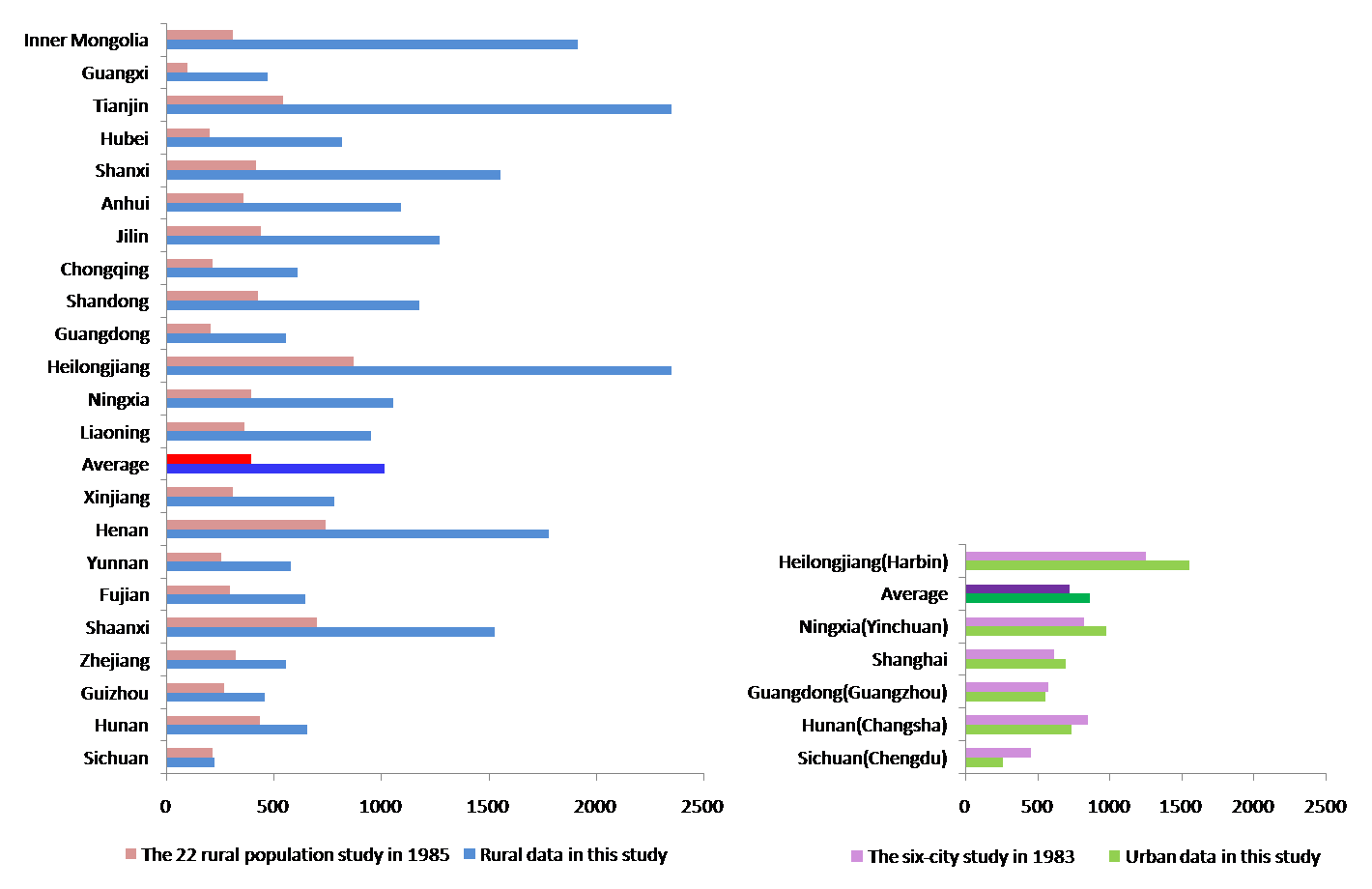
**

Supplemental Fig 2 Comparison of stroke prevalence (age-standardized to the 1960 United States population) with the 22 rural population study and the 6-city stroke study
